# Lack of Association between Insufficient Intake of Multiple Vitamins and Frailty in Older Adults Who Consume Sufficient Energy and Protein: A Nationwide Cross-Sectional Study

**DOI:** 10.3390/nu16162586

**Published:** 2024-08-06

**Authors:** Seung-Guk Park, Hyoeun Kim

**Affiliations:** Department of Family Medicine, Inje University Haeundae Paik Hospital, Busan 48108, Republic of Korea; sgpark@paik.ac.kr

**Keywords:** frailty, proteins, energy intake, vitamins, geriatrics

## Abstract

Frailty is a complex condition that intensifies with age and is marked by decreased physiological function. We rigorously investigated the effects of lower vitamin intake on frailty using data from 665 adults aged over 65 years who consumed sufficient recommended daily energy and protein intakes from the Korean Nutrition and Health Survey, 2016–2019. The definition of frailty was modified based on Fried et al.’s definition of weight loss, exhaustion, weakness, slowness, and low energy expenditure. Based on daily intake, we analyzed vitamins such as vitamin A, thiamine, riboflavin, niacin, folic acid, and vitamin C. Our results of logistic regression showed that increasing multiple deficiencies in several kinds of vitamins (mild to moderate to severe) is not associated with frailty (odds ratio: 1, 1.24 (0.24–3.10), 0.82 (0.28–2.39), *p* for trend = 0.626) in older adults who consumed sufficient calories and proteins. A subgroup analysis of age and sex, which may interfere with the relationship between vitamin intake and frailty, showed that vitamin intake was not associated with frailty when sufficient energy and proteins were consumed. Furthermore, there was no difference in the prevalence of frailty between the groups with sufficient and insufficient intakes of individual vitamins.

## 1. Introduction

As the older population increases, various health problems occur due to aging. Even in older adults without diseases, natural changes may be experienced with age, such as decreased kidney function [1]. However, frailty, a severe deterioration in function that interferes with daily life, differs from normal aging and is a global geriatric health issue [2,3,4,5]. Frailty is more prevalent in older people than in younger people. It refers to a condition in which the physiological functions of the bodily organs decline, and vulnerability to external stress increases, increasing the risk of disease morbidity [3,5]. Older adults with frailty are at high risk of falls, hospitalization, functional decline, fractures, and death, making early diagnosis and intervention crucial [2,3,4,5]. Therefore, preventing and managing the onset and progression of frailty can positively reduce the social and medical burden [6].

The frail population is expected to increase as the older population increases; however, a Korean study using nationally representative data showed that the prevalence of frailty decreased between 2008 and 2020, and the frailty index decreased in all age groups. This is presumed to result from improved nutritional status, chewing, activity, and lifestyle habits [7]. Traditionally, frailty is believed to occur and progress irreversibly with age. However, healthy aging can affect the development and progression of frailty, and recent evidence suggests that frailty progression can be prevented by nutritional support, exercise, or other multidimensional interventions [8,9]. The decreasing trend in frailty reported in a previous study also supports these findings [7].

Nutrition is essential in preventing and managing frailty [5,10,11,12,13]. However, older people are more likely to develop malnutrition because of decreased physiological function resulting from aging, social and psychological isolation, pre-existing comorbidities and medication use, reduced physical activity levels, poor eating habits, and lack of nutritional knowledge [5,10]. Diet is a modifiable factor that can be used to manage frailty [14]. Previous studies have shown that total energy and protein levels are crucial in patients with frailty, and vitamin deficiencies are associated with frailty [10,11,13,15]. Protein intake is closely associated with frailty because it is essential for muscle synthesis [10,11,13]. A diet high in protein indicates a diet with high total energy and nutrient intake [11,13]. However, low energy intake can cause nutritional deficiencies, muscle wasting, and further-advancing frailty [11]. These results are inconsistent; however, insufficient vitamin intake that does not meet the recommended dietary allowance has been linked to frailty, as vitamins can cause frailty and affect muscle formation [5,15].

Notably, few studies have analyzed the relationship between dietary intake and frailty in older adults. However, no study has examined the relationship between frailty and insufficient multiple vitamin intake in groups with sufficient energy and protein intake. Discrepancies in the results regarding the association of vitamin intake with frailty may have been caused by other factors influencing the association. Therefore, we aimed to examine the relationship between frailty and the level of lower vitamin intake in a group with sufficient protein and total energy intakes to rule out confounding factors due to protein and total energy intakes, which are thought to have the most critical influence on nutritional intake.

## 2. Materials and Methods

### 2.1. Data Source and Study Population

The data were analyzed using the 2016–2019 National Health and Nutrition Examination Survey (KNHANES). KNHANES aimed to evaluate the health and nutritional status of Koreans. The survey included health checkups, health interviews, and nutritional surveys. For the analysis, we used height, weight, and grip strength from health examination data, socio-epidemiological information from health interview data, and total energy, macronutrient, and micronutrient intake obtained through a 24 h recall from the nutritional survey data.

Notably, 6691 of the 32,379 participants were aged ≥65 years. Among the 6691 participants, 3752 who had taken nutritional supplements for ≥2 weeks were excluded, and 156 with missing values for frailty and nutrition variables were excluded, resulting in 2784 participants. Among them, 686 met the recommended daily calorie and protein intakes, excluding those who consumed >4000 kcal daily (21 participants). Therefore, the final sample size was 665. The KNHANES 2016–2017 was conducted without a research ethics review. This was because it was government-led research for public welfare based on the opinions of the Research Ethics Review Committee of the Korea Centers for Disease Control and Prevention. This study was approved by the Institutional Review Board of the Inje University Haeundae Paik Hospital (IRB No. 2024-05-015, approved on 20 May 2024).

### 2.2. Definition of Frailty

Fried et al.’s definition of frailty did not precisely match the KNHANES data; therefore, we established a modified definition of frailty by referring to those of previous studies based on the KNHANES data [8,9]. Fried’s five components for diagnosing frailty were as follows: (1) weight loss, defined as an unintentional weight loss of >3 kg in the past year through a self-administered questionnaire; (2) exhaustion, defined as those who answered a questionnaire indicating being highly stressed; and (3) weakness, assessed by grip strength, measured using a digital grip strength dynamometer (TKK 5401; Takei Scientific Instruments Co., Ltd., Tokyo, Japan). According to an Asian working group for sarcopenia, grip strength was <28 kg in men and <18 kg in women [16]. It also included (4) slowness, defined as having a problem with walking or sleeping in the mobility section of the Euro Quality of Life-5 Dimension (EQ-5D) questionnaire; and (5) low energy expenditure, determined by calculating the metabolic equivalent task (MET) using The Global Physical Activity Questionnaire, and based on a previous study that proposed a standard for the physical activity level of the bottom 20% of Korean seniors. The MET score was <494.65 kcal weekly for men and <283.50 kcal weekly for women [17]. Frailty was classified as robust, pre-frail, or frail. Robust was defined as not satisfying any of the five factors; pre-frail was defined as satisfying one or two factors; and frail was defined as satisfying three or more factors. For this analysis, the robust and pre-frail groups were combined to create a non-frail group compared with the frail group.

### 2.3. Nutrient Intake and Classification

In the KNHANES, total calorie, macronutrient, and micronutrient intakes obtained from food were evaluated using the 24 h recall method. The study participants consumed the recommended daily energy and protein intake based on the 2020 Korean dietary standard intake data [18]. The recommended energy amount is ≥2000 kcal daily for men aged 65–74 years, ≥1900 kcal daily for men aged >75 years, ≥1600 kcal daily for women aged 65–74 years, and ≥1500 kcal per day for women aged ≥75 years. The appropriate daily protein intake was ≥60 g for men aged ≥65 years and ≥50 g for women aged ≥65 years.

The nutritional survey suggested the intake levels of eight types of vitamins: vitamin A, thiamine, riboflavin, niacin, folic acid, vitamin C, vitamin D, and vitamin E. However, the recommended nutrient intake (RNI) values for vitamins D and E were not provided, and only their adequate intake (AI) was provided; therefore, they were excluded from the vitamins included in the analysis, and the remaining six were used. 

Vitamin intake status was assigned a score of 0 for satisfactory and 1 for insufficient intake by comparing the RNI values for each vitamin based on age and sex. If there was a satisfactory intake of all vitamins or insufficient intake of one of the vitamins, the degree of vitamin deficiency was set to mild. If two or four vitamins were insufficient, the degree of deficiency was considered moderate. Furthermore, if five or more vitamins were insufficient, the degree of deficiency was considered severe. 

The RNI standards for each vitamin recommended for older adults are as follows. Vitamin A μg retinol equivalent/day (65–74 y: ≥510, ≥75 y: ≥500 in men; 65–74 y: ≥410, ≥75 y: ≥410 in women), thiamine mg/day (65–74 y: ≥1.1, ≥75 y: ≥1.1 in men; 65–74 y: ≥1.0, ≥75 y: ≥0.8 in women), riboflavin mg/day (65–74 y: ≥1.4, ≥75 y: ≥1.3 in men; 65–74 y: ≥ 1.1, ≥75 y: ≥1.0 in women), niacin mg niacin equivalent/day (65–74 y: ≥14, ≥75 y: ≥13 in men; 65–74 y: ≥13, ≥75 y: ≥12 in women), folate μg dietary folate equivalent/day (65–74 y: ≥400, ≥75 y: ≥400 in men; 65–74: ≥400, ≥75 y: ≥400 in women), and vitamin C mg/day (65–74 y: ≥100, ≥75 y: ≥100 in men; 65–74 y: ≥100, ≥75 y: ≥100 in women).

### 2.4. Sociodemographic Variables

The age ranges of the participants were 65–74 years and over 75 years, and their education level was divided into middle school graduation or less (≤9 years) and high school graduation or higher (>9 years). Household income was divided into four quartiles and reclassified into two groups: <25% and ≥25%. Residence status was categorized as living together or alone, and smoking status was categorized as a current smoker or others (former smoker and non-smoker). The frequency of drinking was divided into less than once weekly and more than twice or thrice weekly. Physical activity was assessed using MET values. Based on the Global Physical Activity Questionnaire, a person with a physical activity level in the lower 20% of the older population in Korea according to sex was defined as having a low MET. Body mass index (BMI) was divided into four groups according to Asian standards: underweight, normal weight, overweight, and obese [19]. The diagnosis of cancer and chronic diseases was confirmed through a health interview, and out of 14 diseases, they were classified into a group with three or more chronic medical diseases and a group with fewer than three diseases. The types of cancer and chronic diseases evaluated as comorbidities included breast, liver, stomach, colon, cervical, lung, thyroid, and other cancers, as well as hypertension, angina pectoris, myocardial infarction, diabetes, stroke, and chronic kidney disease. 

### 2.5. Statistical Analyses

The KNHANES has a complex sample design that represents the Korean population. It was analyzed by applying weights based on variables and years. General linear model analysis was performed on continuous variables, and chi-square analysis was performed on categorical variables to compare variables between the frail and non-frail groups. We performed a multivariate logistic analysis to determine whether there was a difference in the frequency of frailty between groups with different degrees of insufficient vitamin intake and between groups with and without sufficient intake of each vitamin. Age, sex, education level, income level, living status, BMI, drinking frequency, smoking status, and chronic diseases were used as adjustable variables. Subgroups according to age and sex were analyzed using the same method. We used STATA 15.0 SE (Stata Corp., College Station, TX, USA) for statistical analysis, and the significance level was set at *p* < 0.05.

## 3. Results

The proportion of older adults with frailty aged ≥65 years who met the recommended energy and protein intake was 10.98%. Table 1 shows the clinical characteristics of the non-frail group, the frail group, and all the participants. Comparing the frail and non-frail groups, the frail group was older and had a higher proportion of women than the non-frail group. They also had a high rate of lower levels of education and household income and a high number of comorbidities. Regarding health-related variables, the two groups showed no difference in the proportions of those living alone, smokers, or frequent drinkers. However, the amount of physical activity assessed by noting that low MET per week was higher in the frail group than in the non-frail group. There was no difference in the average BMI value between the two groups, but the frail group had a higher proportion of people who were underweight and obese than the non-frail group.

The research findings revealed no significant differences in the intake of total energy, macronutrients (carbohydrates, proteins, and fats), or vitamins (vitamin A, thiamine, riboflavin, niacin, folate, and vitamin C) between the two groups. Focusing on each vitamin, only approximately 20% of the participants consumed the recommended amount of vitamins A and C in the non-frail and frail groups, followed by folate and niacin at approximately 50%. However, despite the participants consuming energy and protein above the recommended standards, they had deficient vitamin intakes based on the recommended standards. In the insufficient levels of vitamin intake defined in this study, the proportion of mild cases was 17.5%, 66.8% were moderate, and 15.7% were severe. There was no significant difference in the risk of frailty with an increasing degree of vitamin deficiency, from mild (number of deficient vitamins was zero–one) to severe (number of deficient vitamins was five–six) (Table 2). 

In a subgroup analysis that divided participants into men and women, 65–75 years, and ≥75 years age groups, no significant correlation was observed between the degree of insufficient vitamin intake and frailty (Table 3). Furthermore, when frailty was analyzed by dividing it into frailty components, it was difficult to determine its correlation with insufficient vitamin intake (Table 4). 

Table 5 shows the odds ratio of frailty in the group that did not meet the recommended intake for each vitamin compared with the group that satisfied the recommended intake. However, there were no significant differences in the vitamin levels.

## 4. Discussion

In this study, insufficient multiple vitamin intake was not associated with frailty in older adults who consumed the recommended daily energy and protein intake. Notably, older adults who consume sufficient energy and protein often do not consume sufficient amounts of vitamins. However, intakes of increased numbers of vitamins were not insufficiently associated with frailty. There was also no difference in frailty between groups with sufficient and insufficient intakes of individual vitamins when comparing the risk of frailty between groups whose intake met the RNI criteria and those who did not. Therefore, the lack of an association between lower vitamin intake and frailty in this study may suggest that micronutrient deficiencies are not likely to be a marker for frailty when consuming enough total calories and protein to meet the recommended daily intake.

Nutrition influences healthy aging and the progression of frailty in older adults [5,10,11,12,13,20,21]. However, this study confirmed that it is difficult for all individuals to achieve healthy aging without frailty, even those who consume sufficient energy and protein daily. In a previous study that used the KNHANES [6], the frailty rate of the general population aged ≥65 years was 28.1% (men, 20.6%; women, 33.8%), compared with 10.74% (men, 7.02%; women, 15.22%) in our study. This indicates that the frailty rate in participants who consumed sufficient energy and protein seemed to have decreased slightly. This suggests that, although overall energy and protein intake may help prevent frailty, various unidentified and complex factors may also affect the mechanism of frailty.

In this study, the frail and non-frail groups showed no differences in the mean intakes of total calories, protein, fat, or vitamins. However, despite consuming sufficient energy and protein, older adults in the frail and non-frail groups had high rates of unsatisfactory vitamin intake. Previous studies have shown that micronutrient intake varies with calorie intake and tends to increase proportionally. Obtaining an adequate supply of essential vitamins is difficult when consuming <1500 kcal of energy because dietary food alone cannot compensate for low micronutrient intake [22]. The findings of the European Survey on Nutrition and Aging (SENECA) study, which targeted healthy community-dwelling older adults aged 74–79 years, showed that despite sufficient energy intake of >1900 kcal, many of them still had micronutrient deficiency [12]. Similarly, another study [23] reported insufficient vitamin intake, even in healthy older adults with adequate calorie intakes. The average total energy intake of the participants in this study was approximately 2300 kcal, which was higher than the recommended intake for Koreans and in previous studies. However, the proportions of men and women with insufficient intakes of two or more of the six vitamins included in the study were 81.9% and 83.1%, respectively. 

We conducted this study assuming that a lack of vitamin intake mediates the development of frailty; however, we found no correlation between the level of vitamin intake (insufficient levels of vitamin intake 0–1, 2–4, and 5–6) and frailty. This was inconsistent with the results of previous studies [5,10,13,15,21,24]. Balboa-Castillo et al. found that the group with a poor intake of less than the recommended daily intake of five vitamins had a 2.84 times higher risk of frailty than the group with an adequate intake of eight or more vitamins [5]. Other studies have reported that those consuming fewer than four or more nutrients that did not reach the recommended intake had a higher rate of frailty than those who adequately consumed all nutrients, including proteins, vitamins, and minerals [10,15]. Furthermore, some studies have used women’s health and aging to examine the relationship between vitamin deficiency and frailty through serum concentrations [21,24]. The frequency of frailty was associated with a higher prevalence of vitamin deficiency in the blood [21], and the rate of frailty tends to increase with increasing number of nutrient deficiencies [24]. European cohorts of older adults aged ≥65 years showed a relationship between fat-soluble micronutrient patterns and frailty; however, there was no association with the occurrence of frailty at follow-up [25].

This study found no correlation between individual vitamins and frailty based on whether the recommended intake was met for all six vitamins (vitamin A, thiamine, riboflavin, niacin, folate, and vitamin C). Previous studies evaluating vitamin intake reported mixed results that were either relevant or not [5,10,15,26], and the vitamins that were related were vitamin A [15], C [5,10], D [10], E [5,10], B6 [5,26], and folate [5,10]. 

The discrepancy with previous results may be due to differences in the inclusion and exclusion criteria of study participants, differences in the composition of nutrients included in the study, such as vitamins, minerals, and proteins, and the relationship between vitamins and aging [10,15]. This is thought to be due to the differences in the control variables that affect the relationship. Notably, previous studies corrected for total calories but not for protein intake [5], or neither [21,24]. Even in meaningful studies, the most significant relationship between nutrition and frailty disappeared or weakened after adjusting for energy [10,14]. This was due to the significant correlation between the intake of these nutrients and total energy. Our design allows for a more independent assessment of the effects of vitamins by minimizing the impact of protein and energy intake.

Therefore, based on this study, viewing low vitamin intake as a direct risk factor for frailty is difficult when total calorie and protein intake are sufficient. Thus, increasing the total calorie and protein intake before vitamin intake in older adults is recommended as a crucial factor that plays a nutritional role in frailty. Our opinion is supported by the aging management guidelines presented in the Asia-Pacific Clinical Practice Guidelines, which recommend that aging adults be tested for unintended weight loss and its associated reversible causes, and protein and calorie supplementation should be prioritized [27]. In a systematic review, protein and energy supplementation tended to be effective only in malnourished older adults, and the effect of micronutrient supplementation on frailty was limited, similar to our conclusion [28]. Frailty is not caused by a single factor, but by multiple factors, and vitamin intake, compared with protein or energy intake, is predicted to be an insignificant factor that affects this process organically. However, further studies are required in this area.

A previous study suggested that using oral nutritional supplements alone was ineffective, and physical performance significantly improved when this was combined with exercise and dietary interventions [29]. Our study showed that physical activity was noticeably lower in the frail group than in the non-frail group. This is an inevitable difference due to the inclusion of physical activity as a component defining frailty. This cross-sectional study could not determine whether physical activity declined due to frailty or whether a decline in physical activity caused frailty; however, the frail group likely consumed nutrients without exercise training because they could not engage in active physical activity. Similar to the results of previous studies [29], micronutrient consumption without physical activity may not affect the development of frailty.

This study has some limitations. First, only six vitamins were used among the various micronutrients; therefore, micronutrients other than those presented in this study (such as vitamin B6 and D, and minerals) may have affected frailty. Also, this study could not consider interactions between different vitamins and other nutrients that may affect absorption and efficacy. Second, total calories and protein were calculated based on absolute values rather than per kg of body weight; therefore, there may be differences in whether they are supplied insufficiently or more than required depending on body weight. Third, the 24 h dietary recall method used in our study is an indirect method to estimate nutrient intake, so it may have some limitations in evaluating established deficiency states. However, it is widely used as a valid tool to evaluate energy and nutrients, and it especially shows satisfactory results in obtaining nutritional information at the group level. Finally, it was difficult to confirm causality because this was a cross-sectional study. To our knowledge, no previous studies have examined the relationship between frailty and vitamin intake, targeting only groups that satisfied the total calorie and protein intakes. Previous studies on vitamins and frailty are limited, designed for more heterogeneous populations, and lack the necessary statistical adjustments for the relevant covariates. We overcame this problem by studying the KNHANES, a large representative dataset. We determined the influence of total calories and protein, which significantly impact frailty, and controlled for variables that could significantly influence the analysis. We also performed subgroup analyses for age and sex to include additional information on factors that may interfere with the relationship between vitamin intake and frailty. Additionally, individuals who received dietary supplements were excluded from the study to rule out the influence of vitamins and minerals that may have been supplied through the supplements.

## 5. Conclusions

There is lack of evidence to support the development and progression of frailty by consuming a balanced diet of vitamins in Korean older adults consuming sufficient amounts of calories and protein. However, the importance of nutrition in frailty continues to be emphasized. This study confirmed that the insufficient intake of multiple vitamins is common even when energy and protein are consumed satisfactorily. However, to ensure overall well-being in older adults, efforts are needed to adopt a multidisciplinary approach, including a protein-rich diet, the correction of micronutrient deficiencies, and physical activity. Further well-designed studies are required to elucidate the pathogenesis of frailty and the role of vitamins in older adults.

## Figures and Tables

**Table 1:** General characteristics of participants.

Characteristics	Total (*n* = 665)	Non-Frail (*n* = 592)	Frail (*n* = 73)	*p*-Value
Age, years	72.0 (0.2)	71.7 (0.2)	74.5 (0.6)	<0.001
65–74 years, %	67.5 (2.0)	70.6 (2.1)	41.0 (6.8)	<0.001
≥75 years, %	32.5 (2.0)	29.4 (2.1)	59.0 (6.8)	
Sex, women, %	45.3 (2.1)	43.1 (2.2)	64.3 (6.1)	0.002
Education level ≤9 years, %	66.5 (2.5)	63.8 (2.7)	88.9 (4.1)	<0.001
The lowest 25% quartile of income, %	40.0 (2.5)	38.3 (2.6)	54.5 (6.7)	0.021
Living alone, %	17.6 (1.7)	16.9 (1.7)	23.5 (5.1)	0.172
Current smoker, %	10.4 (1.4)	10.4 (1.4)	10.9 (4.3)	0.903
Frequent alcohol drinking, %	25.3 (2.0)	25.6 (2.2)	22.2 (5.2)	0.559
Low total MET per week,%	59.8 (2.5)	55.8 (2.6)	92.9 (3.8)	<0.001
Body mass index, kg/m^2^	24.3 (0.1)	24.3 (0.1)	24.3 (0.4)	0.935
Underweight (BMI < 18.5), %	0.6 (0.3)	0.3 (0.2)	3.4 (1.9)	0.038
Normal (18.5 ≤ BMI < 23), %	33.8 (2.3)	34.1 (2.4)	31.4 (6.2)	
Overweight (23 ≤ BMI < 25), %	25.1 (2.0)	25.3 (2.1)	23.5 (5.9)	
Obesity (25 ≥ besi, %	40.4 (2.4)	40.3 (2.5)	41.7 (6.4)	
Number of comorbidities ≥ 3, %	13.4 (1.5)	11.8 (1.6)	26.5 (5.6)	0.002

MET, metabolic equivalent task; BMI, body mass index. The numbers were unweighted number of observations. The data were expressed as the estimated means ± standard error or estimated percentage. The *p*-values were for the chi-square test for categorical variables and *t*-test for continuous variables between non-frail and frail groups.

**Table 2:** Nutrition information of participants according to frail status.

Characteristics	Total (*n* = 665)	Non-Frail (*n* = 592)	Frail (*n* = 73)	*p*-Value
Nutritional intake per day				
Total energy, kcal	2361.4 (22.5)	2372.0 (23.3)	2274.0 (62.6)	0.133
Carbohydrate, g	388.3 (4.8)	389.6 (5.0)	377.4 (14.4)	0.422
Protein, g	82.6 (1.0)	83.1 (1.1)	79.0 (3.6)	0.274
Fat, g	42.0 (1.1)	42.0 (1.1)	41.9 (4.8)	0.985
Vitamin A, μg REs	439.1 (24.9)	422.2 (17.3)	579.2 (178.1)	0.381
Meet RNI, %	16.7 (1.7)	16.2 (1.7)	20.6 (5.8)	
Thiamine, mg	1.62 (0.03)	1.62 (0.03)	1.66 (0.13)	0.770
Meet RNI, %	87.5 (1.6)	87.6 (1.7)	86.1 (4.7)	
Riboflavin, mg	1.70 (0.03)	1.70 (0.03)	1.63 (0.12)	0.554
Meet RNI, %	74.3 (2.0)	74.1 (2.2)	75.9 (5.2)	
Niacin, mg NEs	15.08 (0.3)	15.16 (0.3)	14.46 (1.0)	0.502
Meet RNI, %	56.6 (2.2)	56.4 (2.4)	58.1 (6.5)	
Folate, μg DFEs	434.5(8.5)	439.8 (8.9)	390.9 (24.3)	0.059
Meet RNI, %	51.6 (2.1)	52.7 (2.2)	42.5 (6.6)	
Vitamin C, mg	74.7 (3.5)	76.0 (3.8)	63.8 (6.7)	0.112
Meet RNI, %	21.1 (2.0)	21.5 (2.1)	18.1 (5.2)	
Insufficient levels of vitamin intake				
Mild, %	17.5 (1.7)	18.2 (1.8)	12.2 (4.4)	0.496
Moderate, %	66.8 (2.1)	66.1 (2.3)	72.4 (5.7)	
Severe, %	15.7 (1.7)	15.7 (1.8)	15.4 (4.3)	

REs, retinol equivalents; RNI, recommended nutritional intake; NEs, niacin equivalents; DFEs, dietary folate equivalents. The numbers are the unweighted number of observations. The data are expressed as the estimated means ± standard error or estimated percentages. The *p*-values were for the chi-square test for categorical variables and the *t*-test was for continuous variables between the non-frail and frail groups. The insufficient level of vitamin intake was given a score of 0 if satisfied and 1 if insufficient by comparing the RNI value for each vitamin according to age. If the total score was 0 or 1, it was mild; if it was 2 to 4, it was moderate; and if it was 5 or more, it was severe.

**Table 3:** Association between insufficient intakes of multiple vitamins and frailty.

	Frail			
	Crude	Model 1	Mode 2	Mode 3
Total				
Mild	1	1	1	1
Moderate	1.62 (0.68–3.87)	1.43 (0.59–3.47)	1.19 (0.48–2.92)	1.24 (0.49–3.10)
Severe	1.46 (0.52–4.05)	1.06 (0.36–3.12)	0.91 (0.30–2.68)	0.82 (0.28–2.39)
*p* for trend	0.434	0.956	0.805	0.626
Male				
Mild	1	1	1	1
Moderate	1.83 (0.60–5.55)	1.39 (0.43–4.48)	1.19 (0.36–3.88)	1.36 (0.35–5.26)
Severe	2.08 (0.46–9.26)	1.64 (0.35–7.66)	1.62 (0.34–7.76)	1.22 (0.21–7.08)
*p* for trend	0.300	0.525	0.550	0.819
Female				
Mild	1	1	1	1
Moderate	1.56 (0.47–5.12)	1.44 (0.43–4.76)	1.14 (0.34–3.81)	1.08 (0.32–3.66)
Severe	1.04 (0.27–4.02)	0.89 (0.22–3.57)	0.71 (0.18–2.82)	0.59 (0.15–2.34)
*p* for trend	0.989	0.776	0.524	0.337
65–74 years				
Mild	1	1	1	1
Moderate	1.04 (0.29–3.67)	1.09 (0.31–3.85)	0.89 (0.24–3.26)	0.83 (0.22–3.12)
Severe	1.34 (0.32–5.50)	1.10 (0.24–4.96)	0.92 (0.20–4.18)	0.75 (0.16–3.48)
*p* for trend	0.692	0.891	0.927	0.718
≥75 years				
Mild	1	1	1	1
Moderate	2.09 (0.62–6.99)	2.01 (0.59–6.83)	1.71 (0.49–6.00)	2.03 (0.57–7.18)
Severe	1.48 (0.33–6.54)	1.30 (0.27–6.06)	1.01 (0.20–4.95)	0.88 (0.18–4.12)
*p* for trend	0.649	0.813	0.862	0.592

Multivariate logistic regression adjusted for Model 1 (age and sex), Model 2 (Model 1 + education level, income level, and living alone), and Model 3 (Model 2 + body mass index, frequency of drinking, smoking status, and presence of chronic disease).

**Table 4:** Association between insufficient intakes of multiple vitamins and frailty components.

	Insufficient Levels of Vitamin Intake			
	Mild	Moderate	Severe	*p* for Trend
Frail	1	1.24 (0.49–3.10)	0.82 (0.28–2.39)	0.626
Frail component				
Unintentional weight loss	1	1.21 (0.58–2.51)	0.60 (0.23–1.55)	0.302
Weakness	1	0.95 (0.49–1.85)	0.63 (0.25–1.60)	0.325
Exhaustion	1	0.55 (0.16–1.86)	0.21 (0.01–2.73)	0.171
Slowness	1	0. 91(0.52–1.59)	0.97 (0.49–1.92)	0.945
Low physical activity	1	1.24 (0.74–2.06)	1.15 (0.56–2.32)	0.674

Multivariate logistic regression adjusted for Model 3 (age, sex, education level, income level, living alone, body mass index, frequency of drinking, smoking status, and presence of chronic disease).

**Table 5:** Odds ratio for the association between adherence of RNI for vitamins and frailty.

	Frail					
	Vitamin A	Thiamine	Riboflavin	Niacin	Folate	Vitamin C
Meet RNI	1	1	1	1	1	1
NOT meet RNI	0.55 (0.26–1.17)	1.34 (0.58–3.09)	0.66 (0.35–1.24)	0.62 (0.34–1.14)	1.19 (0.65–2.17)	0.92 (0.42–2.03)
*p* value	0.125	0.479	0.200	0.127	0.555	0.850

RNI, recommended nutritional intake. Multivariate logistic regression adjusted for Model 3 (age, sex, education level, income level, living alone, body mass index, frequency of drinking, smoking status, and presence of chronic disease).

## Data Availability

The data analyzed in this study are public resources and can be accessed through the following link: (https://knhanes.kdca.go.kr/knhanes/sub04/sub04_01.do?classType=1, accessed on 10 July 2024). The instructions for accessing raw data and information are available only in Korean.
